# Editorial Does the Scientific Community Need Another Open-Access Journal?

**DOI:** 10.1109/OJEMB.2020.2969731

**Published:** 2020-07-24

If you are looking at the first papers published in the IEEE Open Journal of Engineering in Medicine and Biology (OJEMB) and you are wondering if the scientific community truly needed another open-access journal, you are not alone. I asked myself the same question when I was approached to serve as the Founding Editor-in-Chief of IEEE OJEMB. In fact, over the past two decades, we have witnessed the launch of a multitude of open-access journals, often lacking a well-defined scope and focus on publishing high-quality manuscripts.

Do not get me wrong, I am an enthusiastic supporter of gold open-access journals, but only of those that are meant to address an unmet need of the scientific community and that prioritize quality over collecting article processing charges. Unfortunately, that has not been the case for too many open-access journals. So, when I was asked to consider the opportunity to serve as the Founding Editor-in-Chief of IEEE OJEMB, I asked myself if there was an emerging community of researchers who needed a new open-access journal. Clearly my answer was that such community does exist and that it deserves to have a new journal as its “home.”

This community is made up of scientists, engineers, and clinicians who work at the intersection of biology and technology with the objective of advancing the field of medicine. A portion of this community publishes primarily in journals such as Science and Nature. These journals have primary focus on scientific discoveries and hence use a format in which the *Results* section is presented immediately after the *Introduction* of the manuscript. Another portion of this community is accustomed to publishing in IEEE journals and the like. These journals use a format that puts emphasis on a detailed description of the technology developed by the investigators, which can be found in the *Materials and Methods* section immediately following the *Introduction* of the manuscript.

To facilitate bringing together researchers who typically publish their work in *Science* and *Nature* and researchers who typically publish their work in IEEE journals and the like, we decided to offer what we refer to as a “dual format.” Accordingly, authors can use the Science Manuscript template or the Technology Manuscript template for their submissions. The Science Manuscript template allows the authors to focus primarily on their scientific discoveries. The Technology Manuscript template puts more emphasis on the development of the technology. Both formats are relatively short in length (i.e., the main body of the manuscript is limited to 3,000 words). However, authors can provide readers with additional information in the *Supplementary Materials* section, which is limited to 4,000 words but can include up to 10 additional display items (i.e., figures and tables).

The scientific community we seek to serve demands a journal that provides a rapid review process. Too many times, because of a lengthy review process, our manuscripts can be accessed by our colleagues only months and months after we have finalized the preparation of the material. To address this problem, we have assembled a group of nearly 150 individuals who contribute to IEEE OJEMB as members of the Advisory Board, as Associate Editors, and as members of the Editorial Board. The role of the Advisory Board is to determine the journal's path forward. In contrast, the Associate Editors and the Members of the Editorial Board are solely focused on assuring a rapid review process. So far, the time from submission to first decision has been on average 20 days. As we move forward, we will strive to continue to ensure a rapid review process to make high-quality papers available to the scientific community in a timely manner.

We are also aware of the fact that our community does not need just more papers; it needs more high-quality papers. Hence, we plan to publish only manuscripts that are exceptionally innovative and that are expected to have a major impact in the field. We anticipate that this choice will result in a high rejection rate. To be fair to the authors, we have adopted a two-step review process. First, we perform a preliminary review to establish if the material appears to have the above-stated characteristics. If not, the manuscript is immediately rejected (i.e., within a few days). If the submitted manuscript passes this first preliminary review, then a detailed review is carried out over the following two to three weeks.

The first papers published in this inaugural issue of IEEE OJEMB provide a sample that well represents the broad spectrum of scientific areas that we intend to cover in IEEE OJEMB. It also well represents the high scientific quality that we expect to distinguish manuscripts published in this journal. These papers span topics such as cancer therapy, new ultrasound technology, sensing technology to identify motor phenotypes in Parkinson's disease, and soft robotics. These manuscripts are all marked by highly innovative ideas and the potential for achieving a major impact in the field.

I would like to take the opportunity to thank Shankar Subramaniam, Nigel Lovell, and Metin Akay, who serve as President, Past-President, and President-Elect of IEEE EMBS, respectively. Their suggestions and constant advice were key in the process of launching IEEE OJEMB. I am also in debt to individuals such as Don Ingber, Gilda Barabino, Gordana Vunjak-Novakovic, Jim Collins, Lynn Rochester, Michael Miller, Nicholas Peppas, and Robert Langer who were among the first members of the IEEE OJEMB Advisory Board. Their unconditional support and encouragement to work toward establishing IEEE OJEMB as a highly selective, top-notch journal at the intersection of biology, technology, and medicine made it possible to launch the journal with an outstanding group of papers.

I look forward to working with all of you who strive to advance the field of medicine by pursuing research at the intersection of biology and technology, research that leads to new solutions to challenging clinical problems. IEEE OJEMB is your home!!


Paolo Bonato, Ph.D., Editor-in-Chief


